# Depression, Anxiety and Stress among School-going Adolescents of a Secondary School: A Descriptive Cross-sectional Study

**DOI:** 10.31729/jnma.8067

**Published:** 2023-03-31

**Authors:** Sabina Shrestha, Rajan Phuyal, Pratikshya Chalise

**Affiliations:** 1 Department of Paediatrics, Kathmandu Medical College and Teaching Hospital, Sinamangal, Kathmandu, Nepal; 2 Department of Psychiatry, Kathmandu Medical College and Teaching Hospital, Sinamangal, Kathmandu, Nepal

**Keywords:** *adolescent*, *anxiety*, *depression*, *stress*

## Abstract

**Introduction:**

Mental health of adolescents can affect growth and development, decrease school performance, and impair social relationships with peers and families. The COVID-19 pandemic has changed the social and educational scenario and affected the psychological condition of children and adolescents. This study aimed to find out the prevalence of depression, anxiety and stress among school-going adolescents in a secondary school.

**Methods:**

A descriptive cross-sectional study was done among school-going adolescents of a school from 1 October 2021 to 31 November 2021. Ethical approval was taken from the Institutional Review Committee (Reference number: 0609202101). Data was collected using a questionnaire consisting of sociodemographic parameters and a diagnosis of depression anxiety and stress was made using a standard scale. The whole sampling method was used. Percentage and frequency were calculated for binary data.

**Results:**

Among 95 patients, the depression was found in 31 (32.63%), anxiety in 36 (37.89%), and stress in 3 (3.16%).

**Conclusions:**

The prevalence of depression, anxiety and stress was lower than in other studies done in similar settings. The mental health status of school-going adolescents should be identified and appropriate timely interventions need to be taken. Family members, teachers and the concerned authorities should give emphasis to the psychological well-being of the adolescents.

## INTRODUCTION

Depression is one of the most common illnesses worldwide, with an estimated 3.8% of the population affected. It has resulted from a complex interaction of different social, psychological, and biological factors.^1^ During the coronavirus disease pandemic, being isolated at home for long periods, not getting to meet friends, reduced social interactions, uncertainty about the future, both short and long term, as well as a continuous state of fear of being infected, all these might have led to stress, anxiety and feeling of helplessness in all.^2,3^

Among the global burden of disease and injury in children aged 10-19 years, mental health conditions account for 16%.^4^ Anxiety, depression, post-traumatic stress disorder, sleep disturbances, increased anxiety for self and others, psychological distress, social isolation, and interruption of study are some of the potential psychosocial impacts of COVID-19 in children and adolescents. An adolescent might also suffer from domestic violence, child abuse, child labour, child trafficking, child marriage, sexual exploitation and even death.^5-7^

This study aimed to find out the prevalence of depression, anxiety and stress among school-going adolescents in a secondary school.

## METHODS

This descriptive cross-sectional study was carried out among adolescents of grades 8, 9 and 10 in a conveniently selected school in Kathmandu valley using an online survey from 1 October 2021 to 31 November 2021. Ethical approval was taken from the Institutional Review Committee from Kathmandu Medical College and Teaching Hospital (Reference number: 0609202101). All the students of grade 8, 9 and 10 willing to participate were included in the study. None of the students showed their unwillingness to participate in the study. The whole sampling technique was used and all 95 students during the study period were included in the study.

Data was collected using a valid standard tool called Depression, Anxiety and Stress Scale 21 (DASS-21).^8^ DASS-21 are a set of three self-report scales designed to measure the emotional states of depression, anxiety and stress. Each of the three subscales contains seven items with similar content. Necessary instructions were provided before administering the questionnaire by the use of the school administration's Zoom app by which their regular classes were continued. The data collection tool was dropped to the assignment section of each class. Data were collected using google forms.

Data were entered and analysed using IBM SPSS Statistics 20.0. Frequency and percentages were calculated for binary variables.

## RESULTS

Among 95 students, the depression was seen in 31 (32.63%), anxiety in 36 (37.89%), and that stress in 3 (3.16%). Among 31 students with depression, 11 (11.58%) had mild depression whereas 20 (21.05) had moderate depression. Similarly, mild, moderate, severe and highly severe anxiety was found in 9 (9.47%), 17 (17.89%), 9 (9.47%), and 1 (1.05%) respectively and mild stress was seen in 2 (2.11%) (Table 1).

**Table 1:** Prevalence of depression, anxiety and stress using DASS-21 among school students (n= 95).

Classification	n (%)
**Depression**	
Mild	11 (11.58)
Moderate	20 (21.05)
Severe	-
Extremely severe	-
**Anxiety**	
Mild	9 (9.47)
Moderate	17 (17.89)
Severe	9 (9.47)
Extremely severe	1 (1.05)
**Stress**	
Mild	2 (2.11)
Moderate	1 (1.05)
Severe	-
Extremely severe	-

Depression, anxiety and stress among males was 17 (54.84%), 20 (55.56%) and 2 (66.67%) respectively (Table 2).

**Table 2:** Gender distribution of students with depression, anxiety and stress.

Gender	Depression	Anxiety	Stress
Male	17 (54.84)	20 (55.56)	2 (66.67)
Female	14 (45.16)	16 (44.44)	1 (33.33)
Total	31 (100)	36 (100)	3 (100)

Depression was similar among students of grade 9 and 10 that is 13 (41.94%), anxiety was found to be more among students of grade 9 that is 17 (47.22%) and stress was seen more among students of grade 10 that is 2 (66.67%) (Table 3).

**Table 3:** Class-wise distribution of students with depression, anxiety and stress (n= 95).

Class	Depression	Anxiety	Stress
8	5 (16.13)	3 (8.33)	-
9	13 (41.94)	17 (47.22)	1 (33.33)
10	13 (41.94)	16 (44.44)	2 (66.67)
Total	31 (100)	36 (100)	3 (100)

## DISCUSSION

The prevalence of depression was 32.63%, anxiety was 36 (37.89%), and that of stress was 3.16% in our study. The prevalence was found to be lower than another study done in Chandigarh where the prevalence of depression, anxiety and stress was 65.53%, 80.85%, and 47.02%, respectively.^9^ It was also lower than the similar study done in Palestine where the prevalence of depression, anxiety and stress was 89%, 90.7% and 48.1% respectively.^10^ However, it was higher than another study done in China where depression and anxiety were reported among 22.6% and 18.9% of students of similar age groups.^1^

In our study mild and moderate depression was found to be 11.58% and 21.05% whereas, in a similar study done in Bangladesh, mild and moderate depression was reported in 28.6% and 27.9% respectively which was higher than in our study. Similarly, mild, moderate, severe, and extremely severe anxiety in our study was found to be 9.47%, 17.89%, 9.47%, and 1.05% which are lower compared to another study.^7^

In our study, depression was more among males 54.84% than females 45.16% which was similar to the study done in India where depression in males and females was 53.67% and 46.33% respectively.^11^

The limitation of our study is that it was conducted among students of a single school only and it cannot be generalized to all the students of Nepal. Genetic predisposition and family history were not taken into account. It is recommended to conduct studies among more students for more accurate information.

## CONCLUSIONS

The prevalence of depression, anxiety and stress in this study was lower than in other studies done in similar settings. However, mental health status should be identified in school-going adolescents in order to integrate necessary intervention strategies to improve mental health. It is equally important to provide psychosocial support through early and effective interventions in order to prevent depression, anxiety and stress among adolescents and their impact thereafter.
